# Ventriculo-arterial (VA) coupling and fQRS as new selection criteria for primary prevention ICD placement

**DOI:** 10.1186/s40635-024-00642-7

**Published:** 2024-07-08

**Authors:** Nathan Engstrom, Hayley L. Letson, Kevin Ng, Geoffrey P. Dobson

**Affiliations:** 1 Heart, Sepsis and Trauma Research Laboratory, College of Medicine & Dentistry, James Cook University, 1 James Cook Drive, Townsville, QLD 4811 Australia; 2 Cardiac Investigations, The Townsville University Hospital, 100 Angus Smith Drive, Douglas, QLD 4814 Australia; 3 Cardiology Clinic, Cairns Hospital, 165 Esplanade, Cairns, QLD 4870 Australia

**Keywords:** Fragmented QRS, Heart failure, Ventriculo-arterial coupling, Implantable cardiac defibrillator, Left ventricular ejection fraction, Sudden cardiac death, Arrhythmias

## Abstract

For decades, left ventricular ejection fraction (LVEF < 35%) has been a mainstay for identifying heart failure (HF) patients most likely to benefit from an implantable cardioverter defibrillator (ICD). However, LVEF is a poor predictor of sudden cardiac death (SCD) and ignores 50% of HF patients with mildly reduced and preserved LVEF. The current international guidelines for primary prophylaxis ICD therapy are inadequate. Instead of LVEF, which is not a good measure of LV contractility or hemodynamic characterization, we hypothesize ventriculo-arterial (VA) coupling combined with fragmented QRS (fQRS) will improve risk stratification and patient suitability for an ICD. Quantifying cardiac and aortic mechanics, and predicting active arrhythmogenic substrate, from varying fQRS morphologies, may help to stratify ischemic and non-ischemic patients with different functional capacities and predisposition for lethal arrhythmias. We propose HF patients with a low physiological reserve may not benefit from ICD therapy, whereas those patients with higher reserves and extensive arrhythmogenic substrate may benefit. Our hypothesis combining VA coupling with fQRS changes has the potential to widen HF patient participation (low and high LVEF) and advance personalized medicine for HF patients at high risk of SCD.

## Current guidelines for ICD patient selection are inadequate

Chronic heart failure patients are predisposed to develop ventricular arrhythmias and sudden cardiac death (SCD) [1–4]. Deciding who should receive an implantable cardiac defibrillator (ICD) remains a difficult task. Despite the implantation of over 200,000 ICD devices globally each year, up to 70% of post-implantation deaths *are not attributed to arrhythmic* SCD [2–4]. The most commonly used prognostic tool for primary prophylaxis ICD is left ventricular fraction (LVEF) < 35% together with other HF symptoms [4, 5]. However, low LVEF alone does not predict lethal arrhythmias [4, 5], and SCD has been reported in nearly 40% of cardiovascular deaths in HF patients who have higher preserved LVEF [6, 7] and would not otherwise qualify for ICD assessment [5]. Clearly, the current international guidelines are inadequate and additional prognostic criteria are urgently required to maximize the benefit of ICD therapy.

## LVEF is only part of the answer to ICD selection

LVEF is defined as the LV volume ejected per beat (stroke volume) expressed as a percentage of total ventricular volume (end-diastolic volume) [8, 9]. Although two-dimensional echocardiography or speckle tracking echocardiography remain gold standard measures in the diagnosis, choice of treatment and prognosis of LVEF in HF patients [8], it has a number of shortcomings [9]. First, LVEF is not a measure of intrinsic myocardial contractility [8, 10, 11], and second, it provides little information on the interaction between cardiac performance and the arterial system receiving the blood [1, 9, 12, 13]. Optimal performance requires the heart to pump blood into the vasculature at a rate and volume that matches the capability of the arterial tree to receive it. In short, LVEF fails to provide a “systems approach” to assessing cardiac performance in HF patients and identifying who may benefit from ICD therapy.

## ventriculo-arterial coupling as an improved measure of mechanical performance

**Fig. 1:**
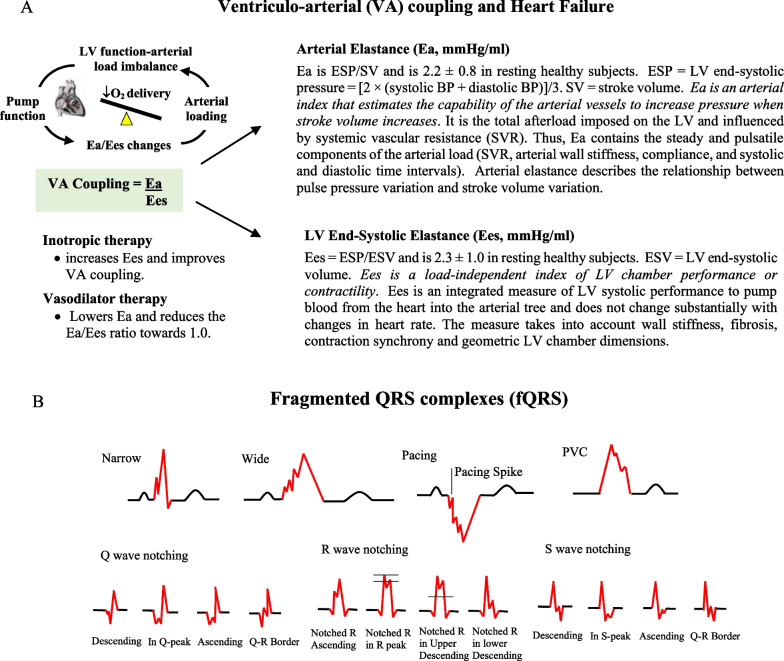
**A** Ventriculo-Arterial (VA) coupling (Ea/Ees) is a measure of mechanical efficiency of heart performance and vascular load function to deliver sufficient O_2_ to the tissues [1, 12, 13]. The function of the arterial system is determined by the relationship between the stroke volume (SV) and end-systolic arterial pressure, where higher SVs lead to higher arterial end-systolic pressures [1, 12, 13]. The slope of this relationship is termed arterial elastance (Ea). Ees is a measure of cardiac contractility and a load-independent index of left ventricular (LV) chamber performance [1, 12, 13]. The advantage of VA coupling over LVEF or cardiac output (CO) is that it provides additional information on arterial loading and left ventricular function. ESP, end systolic pressure; BP, blood pressure. SVR, systemic vascular resistance; ESV, end systolic volume. **B** Different morphologies of fragmented QRS complexes (fQRS) in the 12-lead ECG by Das et al. [18] and modified fQRS Q, R and S criteria after Haukilahti et al. [2]. Modified after Engstrom et al. [20]

A fundamental link between central control, cardiovascular hemodynamics and tissue O_2_ supply is VA coupling [1, 12, 13]. VA coupling is the ratio of arterial elastance (Ea) to left-ventricular (LV) end-systolic elastance (Ees) and provides a measure of how efficiently blood is transferred from the heart to tissue mitochondria (Fig. 1A) [12, 14]. Ea incorporates the elements of arterial load, including peripheral vascular resistance, total arterial compliance, characteristic impedance, and systolic and diastolic time intervals, and Ees is a load-independent index of myocardial contractility and systolic stiffness (Fig. 1). When the Ea/Ees ratio is close to unity, the efficiency of material transfer is considered optimal, meaning that the left ventricle is providing sufficient stroke volume (SV) at its lowest possible myocardial energy consumption. If the ratio is excessively low or high, the heart as a pump and the vascular load become uncoupled and tissue perfusion and O_2_ supply is compromised [12, 15]. In HF patients, as arterial load increases to maintain systolic pressure, Ees decreases and cardiac performance declines, and this leads to VA uncoupling and inefficient contraction [16]. VA coupling in elderly patients with systolic dysfunction has been studied after treadmill exercise by Aslanger and colleagues [16], and in heart failure patients by Antohi and colleagues [17].

We hypothesize that VA coupling, and its components, will be a superior diagnostic and prognostic tool than LVEF for high-risk HF patients because: (1) the index provides a measure of both cardiac and vascular function, including LV functional capacity or physiological reserve; (2) it has the potential to capture all HF patients with low and preserved LVEF; (3) it can be measured using the routine, single-beat, non-invasive echocardiography method of Chen and colleagues [12, 17, 19]; and (4) it can be combined with other measures and risk factors to select patients who are more likely to benefit from ICD therapy.

## Assessing non-viable myocardium and arrhythmogenic substrate

However, VA coupling alone, like LVEF, is insufficient to predict which patients are more likely to die from SCD. We propose combining VA coupling with fragmented QRS (fQRS) from a 12-lead electrocardiogram (ECG) to predict active arrhythmogenic substrate [17]. In a recent review we showed fQRS was associated with ventricular arrhythmias and all-cause mortality in primary prevention HF patients indicated for ICD implantation [3]. fQRS is the zig-zag notching and slurring of the QRS complex that indicates myocardial scarring and fibrosis [2, 20] (Fig. 1B). The size and location of scar or fibrotic region can further be quantified using late gadolinium enhancement cardiac magnetic resonance imaging (Ga-MRI) [4] or myocardial perfusion-gated scintigraphy (SPECT) [21]. Moreover, the different forms of fQRS in HF patients with ischemic cardiomyopathy (ICM) and non-ischemic cardiomyopathy (NICM) may be useful to predict different left ventricular remodelling, conduction defects and active arrhythmogenic substrate [2, 20], which may also be used for personalization of treatment. In summary, we hypothesize VA coupling and fQRS in high-risk HF patients may provide a superior prognostic measure of (1) cardiovascular function; (2) active arrhythmogenic substrate; and (3) SCD, compared to LVEF and HF symptoms.

## Testing the hypothesis

The VA coupling-fQRS hypothesis could be tested in an observational study or prospective, randomized trial using the existing population of ICM and NICM patients with an ICD (LVEF < 35%). The study group should not have experienced a cardiac arrest and already receives routine standard-of-care If suitable, each patient will undergo additional echocardiographic measurements after treadmill exercise tests and stratified into different groups with different functional reserves and scar tissue characteristics [16]. Patient stratification includes using metabolic equivalents (METs), VA coupling, fQRS, Ga-MRI data, New York Heart Association (NYHA) classification and LVEF measurements. We hypothesize that chronic HF patients with low functional capacity (i.e. operating on a more flattened Frank Starling Curve) and minimal scar tissue (i.e. absence of fQRS) will not benefit from ICD therapy. We consider this group at lower risk of triggering severe ventricular arrhythmias [22, 23].

In contrast, we predict that HF patients with higher cardiac reserves (higher scope for activity) and the presence of fQRS (presence of scar tissue) will be more prone to enhanced automaticity, triggered activity and reentry and would benefit from an ICD. Following ICD guidelines, patients recruited in the trial will be monitored every 6 months over a 5-year period, and the study will be powered to include investigating sex-specific differences. Follow-up trials would include patients with low LVEF (< 35%), mid-range LVEF (40–49%) and preserved LVEF (≥ 50%). The latter would be of great interest because ~ 50% of HF patients worldwide have preserved LVEF [24, 25], and ~ 18% of these patients are reported to have fQRS [26]. This trial study has the potential to advance the field of ICD selection. In addition, the study offers an opportunity to include other ECG measures alongside fQRS, such as long QTc [27] or T-peak to T-end (Tpe), which are markers for SCD in the specific patient populations that may benefit from an ICD.

## Conclusions

Identifying HF patients at high risk for developing fatal arrhythmias remains a major challenge in cardiology. To date, no measurement or marker has demonstrated utility in distinguishing which patient will derive benefit from ICD therapy. LVEF has a number of clinical shortcomings. We propose VA coupling combined with fQRS has the potential to redefine the risk stratification criteria for selecting which HF patients are best suited for ICD therapy and possibly improve outcomes. The combined approach may provide a more precision-based medical assessment for all HF patients compared to today’s highly restrictive and failed LVEF-based method.

## Data Availability

The data will be made available for reasonable requests on which this manuscript is based.

## References

[1] Ikonomidis I, Aboyans V, Blacher J, Brodmann M, Brutsaert DL, Chirinos JA, De Carlo M, Delgado V, Lancellotti P, Lekakis J, Mohty D, Nihoyannopoulos P, Parissis J, Rizzoni D, Ruschitzka F, Seferovic P, Stabile E, Tousoulis D, Vinereanu D, Vlachopoulos C, Vlastos D, Xaplanteris P, Zimlichman R, Metra M (2019). The role of ventricular-arterial coupling in cardiac disease and heart failure: assessment, clinical implications and therapeutic interventions. A consensus document of the European Society of Cardiology Working Group on Aorta and Peripheral Vascular Diseases, European Association of Cardiovascular Imaging, and Heart Failure Association. Eur J Heart Fail.

[2] Haukilahti MA, Eranti A, Kentta T, Huikuri HV (2016). QRS fragmentation patterns representing myocardial scar need to be separated from benign normal variants: hypotheses and proposal for morphology based classification. Front Physiol.

[3] Engstrom N, Dobson GP, Ng K, Letson H (2021). Fragmented QRS is associated with ventricular arrhythmias in heart failure patients: a systematic review and meta-analysis. Ann Noninvasive Electrocardiol.

[4] Klem I, Klein M, Khan M, Yang EY, Nabi F, Ivanov A, Bhatti L, Hayes B, Graviss EA, Nguyen DT, Judd RM, Kim RJ, Heitner JF, Shah DJ (2021). Relationship of LVEF and myocardial scar to long-term mortality risk and mode of death in patients with nonischemic cardiomyopathy. Circulation.

[5] Shah KS, Xu H, Matsouaka RA, Bhatt DL, Heidenreich PA, Hernandez AF, Devore AD, Yancy CW, Fonarow GC (2017). Heart failure with preserved, borderline, and reduced ejection fraction: 5-year outcomes. J Am Coll Cardiol.

[6] Adabag S, Langsetmo L (2020). Sudden cardiac death risk prediction in heart failure with preserved ejection fraction. Heart Rhythm.

[7] Wu S-J, Hsieh Y-C (2022). Sudden cardiac death in heart failure with preserved ejection fraction: an updated review. Int J Arrhythm..

[8] Cikes M, Solomon SD (2016). Beyond ejection fraction: an integrative approach for assessment of cardiac structure and function in heart failure. Eur Heart J.

[9] Rizzello V (2022). Selection of patients eligible for implantable cardioverter defibrillator: beyond left ventricular ejection fraction. Eur Heart J Suppl.

[10] Konstam MA, Abboud FM (2017). Ejection fraction: misunderstood and overrated (changing the paradigm in categorizing heart failure). Circulation.

[11] Ashish K, Faisaluddin M, Bandyopadhyay D, Hajra A, Herzog E (2019). Prognostic value of global longitudinal strain in heart failure subjects: a recent prototype. Int J Cardiol Heart Vasc.

[12] Guarracino F, Baldassarri R, Pinsky MR (2013). Ventriculo-arterial decoupling in acutely altered hemodynamic states. Crit Care.

[13] Monge Garcia MI, Jian Z, Hatib F, Settels JJ, Cecconi M, Pinsky MR (2020). Dynamic arterial elastance as a ventriculo-arterial coupling index: an experimental animal study. Front Physiol.

[14] Dobson GP, Morris JL, Letson HL (2022). Why are bleeding trauma patients still dying? Towards a systems hypothesis of trauma (SHOT). Front Physiol.

[15] Granfeldt A, Letson HL, Hyldebrandt JA, Wang ER, Salcedo PA, Nielson TK, Tonnesen E, Vinten-Johansen J, Dobson GP (2014). Small-Volume 7.5% NaCl adenosine, lidocaine and Mg2+ has multiple benefits during hypotensive and blood resuscitation in the pig following severe blood loss: rat to pig translation. Crit Care Med.

[16] Aslanger E, Assous B, Bihry N, Beauvais F, Logeart D, Cohen-Solal A (2015). Effects of cardiopulmonary exercise rehabilitation on left ventricular mechanical efficiency and ventricular-arterial coupling in patients with systolic heart failure. J Am Heart Assoc.

[17] Antohi EL, Chioncel O, Mihaileanu S (2021). Overcoming the limits of ejection fraction and ventricular-arterial coupling in heart failure. Front Cardiovasc Med.

[18] Das MK (2008). Fragmented wide QRS on a 12-lead ECG: a sign of myocardial scar and poor prognosis. Circ Arrhythm Electrophysiol.

[19] Chen CH, Fetics B, Nevo E, Rochitte CE, Chiou KR, Ding PA, Kawaguchi M, Kass DA (2001). Noninvasive single-beat determination of left ventricular end-systolic elastance in humans. J Am Coll Cardiol.

[20] Engstrom N, Letson HL, Ng K, Dobson GP (2022). Predicting arrhythmias in primary prevention heart failure patients: picking up the fragments. Open Heart.

[21] Ozdemir S, Tan YZ, Colkesen Y, Temiz A, Turker F, Akgoz S (2013). Comparison of fragmented QRS and myocardial perfusion-gated SPECT findings. Nucl Med Commun.

[22] Masarone D, Limongelli G, Rubino M, Valente F, Vastarella R, Ammendola E, Gravino R, Verrengia M, Salerno G, Pacileo G (2017). Management of arrhythmias in heart failure. J Cardiovasc Dev Dis.

[23] Landstrom AP, Dobrev D, Wehrens XHT (2017). Calcium signaling and cardiac arrhythmias. Circ Res.

[24] Mihaileanu S, Antohi EL (2020). Revisiting the relationship between left ventricular ejection fraction and ventricular-arterial coupling. ESC Heart Fail.

[25] Savarese G, Stolfo D, Sinagra G, Lund LH (2022). Heart failure with mid-range or mildly reduced ejection fraction. Nat Rev Cardiol.

[26] Gassanov N, Mutallimov M, Caglayan E, Erdmann E, Er F (2022). ECG as a risk stratification tool in patients with wearable cardioverter-defibrillator. J Cardiol.

[27] Khan HM, Leslie SJ (2019). Risk factors for sudden cardiac death to determine high risk patients in specific patient populations that may benefit from a wearable defibrillator. World J Cardiol.

